# Menelloides C and D, New Sesquiterpenoids from the Gorgonian Coral *Menella* sp

**DOI:** 10.3390/md9091534

**Published:** 2011-09-14

**Authors:** Shih-Yao Kao, Jui-Hsin Su, Tsong-Long Hwang, Jyh-Horng Sheu, Zhi-Hong Wen, Yang-Chang Wu, Ping-Jyun Sung

**Affiliations:** 1 Institute of Marine Biotechnology, National Dong Hwa University, Pingtung 944, Taiwan; E-Mails: sweetcloud0906@gmail.com (S.-Y.K.); x2219@nmmba.gov.tw (J.-H.S.); 2 National Museum of Marine Biology and Aquarium, Pingtung 944, Taiwan; 3 Division of Marine Biotechnology, Asia-Pacific Ocean Research Center, National Sun Yat-sen University, Kaohsiung 804, Taiwan; E-Mails: sheu@mail.nsysu.edu.tw (J.-H.S.); wzh@mail.nsysu.edu.tw (Z.-H.W.); 4 Graduate Institute of Natural Products, Chang Gung University, Taoyuan 333, Taiwan; E-Mail: htl@mail.cgu.edu.tw; 5 Department of Marine Biotechnology and Resources, National Sun Yat-sen University, Kaohsiung 804, Taiwan; 6 Graduate Institute of Integrated Medicine, College of Chinese Medicine, China Medical University, Taichung 404, Taiwan; 7 Natural Medicinal Products Research Center and Center for Molecular Medicine, China Medical University Hospital, Taichung 404, Taiwan; 8 Department of Life Science and Institute of Biotechnology, National Dong Hwa University, Hualien 974, Taiwan

**Keywords:** menelloide, lindenane, germacrane, sesquiterpenoid, *Menella*, elastase

## Abstract

Two new metabolites, including a lindenane-type sesquiterpenoid, menelloide C (**1**), and a germacrane-type sesquiterpenoid, menelloide D (**2**), were isolated from a Formosan gorgonian coral identified as *Menella* sp. The structures of **1** and **2** were established by spectroscopic methods and **2** displayed a weak inhibitory effect on the release of elastase by human neutrophils.

## 1. Introduction

**Figure 1:**
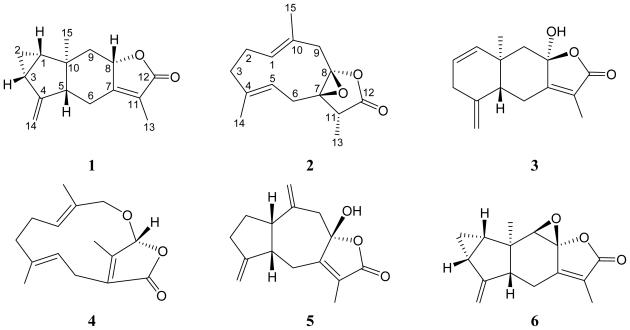
The structures of menelloides C (**1**), D (**2**), (−)-hydroxylindestrenolide (**3**), menelloide A (**4**), menelloide B (**5**), and (+)-chloranthalactone B (**6**).

Previous chemical investigations on gorgonian corals belonging to genus *Menella* (family Plexauridae) [1] have yielded a series of interesting natural products [2–8]. In continuation of our search for new substances from the invertebrates collected off the waters of Taiwan, four new sesquiterpenoid derivatives, (−)-hydroxylindestrenolide (**3**) [9], menelloide A (**4**), menelloide B (**5**), and (+)-chloranthalactone B (**6**) [10] have been isolated from the gorgonian *Menella* sp. We have further isolated two new sesquiterpenoids, including a lindenane-type sesquiterpenoid, menelloide C (**1**), and a germacrane-type sesquiterpenoid, menelloide D (**2**) (Figure 1) from *Menella* sp. In this paper, we describe the isolation, structure characterization, and bioactivity of sesquiterpenoids **1** and **2**.

## 2. Results and Discussion

Menelloide C (**1**) was isolated as a needle solid and the molecular formula for this compound was determined to be C_15_H_18_O_2_ (7° of unsaturation) using HRESIMS (C_15_H_18_O_2_Na, *m/z* 253.1206, calculated 253.1204). The IR spectrum of **1** showed a strong band at 1744 cm^−1^, consistent with the presence of ester group. From the ^13^C NMR data (Table 1), a suite of resonances at δ_C_ 174.8 (C-12), 162.4 (C-7), 122.6 (C-11), 78.4 (CH-8), and 8.6 (CH_3_-13), could be assigned to the α-methyl-α,β-unsaturated-γ-lactone moiety in **1**. An additional unsaturated functionality was indicated by ^13^C NMR resonances at δ_C_ 151.4 (C-4) and 106.6 (CH_2_-14), suggesting the presence of an exocyclic carbon-carbon double bond. On the basis of overall unsaturation data, compound **1** was concluded to be a molecule possessing four rings.

From the ^1^H–^1^H COSY spectrum of **1** (Table 1), it was possible to differentiate between the separate spin systems of H-1/H_2_-2/H-3, H-5/H_2_-6, and H-8/H_2_-9. These data, together with the key HMBC correlations between protons and quaternary carbons of **1**, such as H-2β/C-4; H-6α, H_2_-9, H_3_-13/C-7; H-2β, H-9α, H_3_-15/C-10; H_3_-13/C-11; and H_3_-13/C-12 permitted the elucidation of the carbon skeleton of **1** (Table 1). The exo-cyclic carbon-carbon double bond at C-4 was confirmed by the HMBC correlations between H_2_-14/C-5. The vinyl methyl group at C-11 was established by the HMBC correlations between H_3_-13/C-7, C-11, C-12. The ring junction CH_3_-15 was positioned at C-10 from the HMBC correlations between H_3_-15/C-1, C-5, C-9, C-10 and H_2_-9/C-15. Therefore, the proposed skeleton of **1** was established and suggested to be a lindenane-type sesquiterpenoid.

The relative configuration of **1** was elucidated by a NOESY spectrum which was compatible with those of **1** offered by computer modeling (Table 2), in which the close contacts of atoms calculated in space were consistent with the NOESY correlations. In the NOESY experiment of **1**, H-8 showed correlations with H-5 and H-9β, indicating that these protons were situated on the same face and assigned as β-protons. Furthermore, H_3_-15 showed correlations with H-9α, but not with H-5, suggesting that CH_3_-15 was α-oriented. H-1 exhibited correlations with H-3 and H-9β, indicating that the cyclopropane ring was positioned on the α face in **1**.

**Figure 2:**
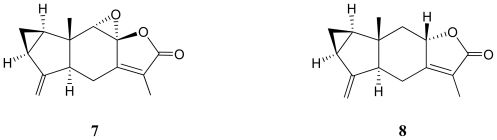
The structures of chloranthalactone B (**7**) and shizukanolide (**8**).

In our previous study, a lindenane-type sesquiterpenoid, (+)-chloranthalactone B (**6**) ([α]^25^_D_ +136 (*c* 0.05, CHCl_3_)), was isolated from this study material *Menella* sp. [10] and this compound was proven to be an enantiomer of a known compound, chloranthalactone B (**7**) ([α] −130.3 (*c* 0.1, MeOH)) (Figure 2), which was isolated from the roots of *Chloranthus glaber* and *Chloranthsu japonicus*, respectively [11–13]. It was found that the structure of **1** was similar to those of lindenanes **6** and **7** except for the 8,9-epoxy group [10–12]. It is interesting to note that the lindenane-type sesquiterpenoids possessing a cyclopropane moiety, presented as structures **1** (menelloide C, ([α]^25^_D_ +57 (*c* 0.04, CHCl^3^)) and **6** ((+)-chloranthalactone B) [10], isolated from *Menella* sp. were suggested to possess the same configurations for the chiral carbons C-5 and C-10 because these two compounds were isolated from the same organisms.

Moreover, the structure of **1** was compared with that of a known sesquiterpenoid metabolite, shizukanolide (**8**) (Figure 2), which was first isolated from a Japanese plant *Chloranthus japonicus* (Chloranthaceae) [14,15]. It was found that these two compounds possessed the same planar structures and **1** was found to be a diastereomer of shizukanolide (**8**) by comparison of the NMR data of **1** with those of **8**.

Compound **2** (menelloide D), obtained as a colorless oil, showed an [M + Na]^+^ signal at *m/z* 271.1312 in the HRESIMS, suggesting the molecular formula C_15_H_20_O_3_ (calcd C_15_H_20_O_3_Na, 271.1310), with 6° of unsaturation. The IR spectrum of **2** showed a band at 1798 cm^−1^, consistent with the presence of γ-lactone group. The ^13^C NMR and DEPT spectra of **2** showed that this compound has 15 carbons (Table 3), including three methyls, four sp^3^ methylenes, an sp^3^ methine, two sp^2^ methines, two sp^3^ quaternary carbons, and three sp^2^ quaternary carbons. From the ^1^H and ^13^C NMR spectra (Table 3), **2** was found to possess a γ-lactone moiety (δ_C_ 175.6, C-12) and two trisubstituted olefins (δ_H_ 4.93, 1H, dd, *J* = 11.0, 5.0 Hz, H-1; δ_C_ 131.3, C-10; 129.6, CH-1; δ_H_ 4.41, 1H, d, *J* = 11.0 Hz, H-5; δ_C_ 130.5, C-4; 121.3, CH-5). The presence of a tetrasubstituted epoxy group was confirmed from the signals of two oxygenated quaternary carbons at δ_C_ 92.8 (C-8) and 71.0 (C-7) and this epoxy group could be a part of a hemiketal constellation in the γ-lactone moiety on the basis of a characteristic carbon signal at δ_C_ 92.8 (C). Thus, from the above data, compound **2** was identified as a tricyclic compound.

From the ^1^H–^1^H COSY spectrum of **2**, three different structural units, C-1/C-2/C-3, C-5/C-6, and C-11/C-13, were identified (Table 3), which were assembled with the assistance of an HMBC experiment (Table 3). The HMBC correlations between protons and quaternary carbons such as H-2α, H-3β, H_2_-6, H_3_-14/C-4; H_2_-6, H-9β, H-11, H_3_-13/C-7; H_2_-6, H_2_-9/C-8; H-2α, H_2_-9, H_3_-15/C-10; and H-11, H_3_-13/C-12 were employed successfully to establish the planar structure of **2**.

The relative stereochemistry of **2** was established on the basis of a NOESY experiment and by vicinal ^1^H–^1^H coupling constant analysis. In the NOESY experiment of **2** (Table 4), correlations observed between H_3_-14 and δ_H_ 2.62 as H-6β; and H_3_-15 and δ_H_ 2.03 as H-2α, as well as the lack of correlation observed between H-1 and H_3_-15 and H-5 and H_3_-14, reflected the *E* geometry of double bonds at C-1/10 and C-4/5. H-5 showed a NOESY correlation with δ_H_ 2.91 as H-6α and no coupling constant (*J* = 0.0 Hz) was found between these two protons indicating the dihedral angle between these two protons is approximately 90° by modeling analysis. H_3_-13 showed a correlation with H-6α, which suggests that H-11 was β-oriented in the γ-lactone moiety. Moreover, there is no correlation between H-11 and any proton in **2** except with H_3_-13. Based on this finding, the epoxy group between C-7/8 should be β-oriented and led to the stereohindrance between H-11 and C-6 methylene protons by modeling analysis.

The *in vitro* anti-inflammatory effects of **2** were tested. Sesquiterpenoid **2** displayed a weak inhibitory effect on the release of elastase by human neutrophils (inhibition rate 10.5%) at a concentration of 10 μg/mL.

## 3. Experimental Section

### 3.1. General Experimental Procedures

Melting points were determined using a Fargo apparatus and were uncorrected. Optical rotations were measured on a Jasco P-1010 digital polarimeter. Infrared spectra were recorded on a Varian Diglab FTS 1000 FT-IR infrared spectrophotometer; peaks are reported in cm^−1^. The NMR spectra were recorded on a Varian Inova 500 NMR spectrometer using the residual CHCl_3_ signal (δ_H_ 7.26 ppm) as an internal standard for ^1^H NMR and CDCl_3_ (δ_C_ 77.1 ppm) for ^13^C NMR. Coupling constants (*J*) are given in Hz. ESIMS and HRESIMS were recorded on a Bruker APEX II mass spectrometer. Column chromatography was performed on silica gel (230–400 mesh, Merck, Darmstadt, Germany). TLC was carried out on precoated Kieselgel 60 F_254_ (0.25 mm, Merck) and spots were visualized by spraying with 10% H_2_SO_4_ solution followed by heating. HPLC was performed using a system comprised of a Hitachi L-7100 pump, a Hitahci L-7455 photodiode array detector, and a Rheodyne injection port. A normal phase column (Hibar 250 × 10 mm, Merck, silica gel 60, 5 μm) was used for HPLC.

### 3.2. Animal Material

Specimens of the gorgonian corals *Menella* sp. were collected by trawling off the coast of southern Taiwan at a depth of 100 m in December 2004 and stored in a freezer until extraction. A voucher specimen (NMMBA-TW-GC-005) was deposited in the National Museum of Marine Biology and Aquarium, Taiwan. This organism was identified by comparison with previous descriptions [1].

### 3.3. Extraction and Isolation

The freeze-dried and minced material of *Menella* sp. (wet weight 451 g, dry weight 411 g) was extracted with ethyl acetate (EtOAc) at room temperature. The EtOAc layer (5.07 g) was separated on silica gel and eluted using *n*-hexane/EtOAc (stepwise from 100:1 to 0:100 *n*-hexane/EtOAc) to yield fractions 1–16. Fraction 3 was separated by normal-phase HPLC (NP-HPLC), using the mixtures of *n*-hexane and EtOAc (15:1–pure EtOAc) to yield the fractions 3A–3Z. Fraction 3H was purified by NP-HPLC using the mixtures of *n*-hexane and acetone (20:1) to afford **2** (1.0 mg). Compound **1** (0.8 mg) was obtained from fraction 3S by NP-HPLC (*n*-hexane/EtOAc, 10:1).

Menelloide C (**1**): needle solid; mp 97–99 °C; ([α]^25^_D_ +57 (*c* 0.04, CHCl_3_); IR (neat) ν_max_ 1744 cm^−1; 1^H (CDCl_3_, 500 MHz) and ^13^C (CDCl_3_, 125 MHz) NMR data, see Table 1; ESIMS: *m/z* 253 [M + Na]^+^; HRESIMS: *m/z* 253.1206 (calcd for C_15_H_18_O_2_ + Na, 253.1204).

Menelloide D (**2**): colorless oil; ([α]^25^_D_ −36 (*c* 0.05, CHCl_3_); IR (neat) ν_max_ 1798 cm^−1; 1^H (CDCl_3_, 500 MHz) and ^13^C (CDCl_3_, 125 MHz) NMR data, see Table 3; ESIMS: *m/z* 271 [M + Na]^+^; HRESIMS: *m/z* 271.1312 (calcd for C_15_H_20_O_3_ + Na, 271.1310).

### 3.4. Molecular Mechanics Calculations

Implementation of the MM2 force field [16] in CHEM3D PRO software from CambridgeSoft Corporation (Cambridge, MA, USA; ver 9.0, 2005) was used to calculate molecular models.

### 3.5. Elastase Release by Human Neutrophils

Human neutrophils were obtained by means of dextran sedimentation and Ficoll centrifugation. Measurements of elastase release were carried out according to previously described procedures [17]. Elastase release experiments were performed using MeO-Suc-Ala-Ala-Pro-Valp-nitroanilide as the elastase substrate.

## 4. Conclusions

In previous studies, a series of interesting natural products, including steroids [4,6,8], guaiane lactones [5,7], briarane diterpenoids [8], menellin A (a highly oxygenated racemate with C8 skeleton) [8], picolinic acid *N*-methyl betaine [3,4], *n*-hexadecanol [4], 9*H*-purin-6-amino-*N*-9-dimethyl [4], thymidine [4], and batyl alcohol [2,4], were isolated from gorgonian corals belonging to genus *Menella*, collected off the South China Sea. In our studies on the chemical constituents of a gorgonian coral identified as *Menella* sp., collected off the waters of Taiwan, various sesquiterpenoids featuring the guaiane, lindenane, and germacrane-type carbon skeletons, containing a γ-lactone in their structures, were isolated. As described in previous studies, the organic extract of *Menella* sp. displayed significant inhibitory effects on the generation of superoxide anion and the release of elastase [9,10]. However, at this stage, the results showed that the compounds that we isolated only showed weak activity. We suggested that the active components are still existed in the other fractions and these fractions will be studied in the future.

## Figures and Tables

**Table 1:** NMR Spectroscopic Data (500 MHz, CDCl_3_) for Menelloide C (**1**).

Menelloide C (1)				
Position	δ_C_, Mult.	δ_H_ (*J* in Hz)	^1^H–^1^H COSY	HMBC
1	28.9, CH	1.38, ddd (7.5, 7.5, 3.5)	2, 3	n.o. a
2α	16.6, CH_2_	0.70, m	1, 2β, 3	n.o.
β		0.84, m	1, 2α, 3	4, 10
3	23.8, CH	2.02, m	1, 2	n.o.
4	151.4, C			
5	56.5, CH	3.02, m	6	n.o.
6α	22.9, CH_2_	2.35, dd (18.0, 12.5)	5, 6β	5, 7, 8
β		2.54, m	5, 6α	n.o.
7	162.4, C			
8	78.4, CH	5.19, m	9	n.o.
9α	43.3, CH_2_	1.82, dd (13.0, 9.0)	8, 9β	5, 7, 8, 10, 15
β		2.62, dd (13.0, 11.5)	8, 9α	7, 8, 15
10	38.9, C			
11	122.6, C			
12	174.8, C			
13	8.6, CH_3_	1.82, s		7, 11, 12
14a	106.6, CH_2_	5.03, s	14b	5
b		4.75, s	14a	5
15	21.2, CH_3_	0.51, s		1, 5, 9, 10

a n.o. = not observed.

**Table 2:** The Stereoview of **1** (Generated from Computer Modeling) and the Calculated Distances (Å) between Selected Protons Having Key NOESY Correlations.

Menelloide C (1)	H/H	(Å)
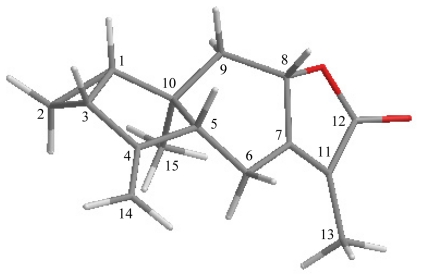	H-1/H-3	2.43
	H-1/H-9β	2.42
	H-5/H-8	2.68
	H-8/H-9β	2.30
	H-9α/H_3_-15	2.58

**Table 3:** NMR Spectroscopic Data (500 MHz, CDCl_3_) for Menelloide D (**2**).

Menelloide D (2)				
Position	δ_C_, Mult.	δ_H_ (*J* in Hz)	^1^H–^1^H COSY	HMBC (H→C)
1	129.6, CH	4.93, dd (11.0, 5.0)	2	2, 9, 15
2α	26.7, CH_2_	2.03, m	1, 2β, 3	1, 3, 4, 10
β		2.12, m	1, 2α, 3	n.o. a
3α	38.9, CH_2_	2.20, ddd (12.0, 3.0, 3.0)	2, 3β	1
β		1.74, ddd (12.0, 12.0, 4.0)	2, 3α	1, 2, 4, 5, 14
4	130.5, C			
5	121.3, CH	4.41, d (11.0)	6	3
6α	25.9, CH_2_	2.91, d (17.0)	5, 6β	4, 5, 7, 8
β		2.62, dd (17.0, 11.0)	5, 6α	4, 5, 7, 8
7	71.0, C			
8	92.8, C			
9α	40.6, CH_2_	3.01, d (14.5)	9β	1, 8, 10, 15
β		3.14, d (14.5)	9α	1, 7, 8, 10, 15
10	131.3, C			
11	43.4, CH	2.72, q (7.0)	13	6, 7, 12, 13
12	175.6, C			
13	10.1, CH_3_	1.36, d (7.0)	11	7, 11, 12
14	17.0, CH_3_	1.59, s		3, 4, 5
15	17.0, CH_3_	1.34, s		1, 9, 10

a n.o. = not observed.

**Table 4:** The Stereoview of **2** (Generated from Computer Modeling) and the Calculated Distances (Å) between Selected Protons Having Key NOESY Correlations.

Menelloide D (2)	H/H	(Å)
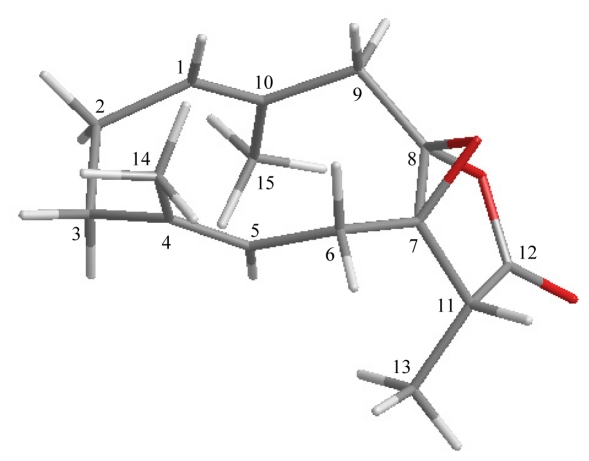	H-2α/H_3_-15	2.50
	H-5/H-6α	2.89
	H-6α/H_3_-13	2.44
	H-6β/H_3_-14	2.45
